# Verapamil Inhibits Mitochondria-Induced Reactive Oxygen Species and Dependent Apoptosis Pathways in Cerebral Transient Global Ischemia/Reperfusion

**DOI:** 10.1155/2020/5872645

**Published:** 2020-10-17

**Authors:** Ehsan Jangholi, Zahra Nadia Sharifi, Mohammad Hoseinian, Mohammad-Reza Zarrindast, Hamid Reza Rahimi, Ashkan Mowla, Hoda Aryan, Mohammad Amin Javidi, Yekta Parsa, Fariborz Ghaffarpasand, Soheila Yadollah-Damavandi, Hamid Zaferani Arani, Farshad Shahi, Shabnam Movassaghi

**Affiliations:** ^1^Department of Neurosurgery, Shariati Hospital, Tehran University of Medical Sciences, Tehran, Iran; ^2^Anatomical Sciences Department, Tehran Medical Sciences, Islamic Azad University, Tehran, Iran; ^3^Young Researchers and Elite Club, Tehran Medical Sciences, Islamic Azad University, Tehran, Iran; ^4^Department of Pharmacology, School of Medicine, Tehran University of Medical Sciences, Tehran, Iran; ^5^Medical Genomics Research Center and School of Advanced Sciences in Medicine, Tehran Medical Sciences, Islamic Azad University, Tehran, Iran; ^6^Department of Medical Genetics and Molecular Medicine, Faculty of Medicine, Mashhad University of Medical Sciences, Mashhad, Iran; ^7^Neurogenic Inflammation Research Center, Mashhad University of Medical Sciences, Mashhad, Iran; ^8^Division of Endovascular Neurosurgery, Department of Neurological Surgery, Keck School of Medicine, University of Southern California (USC), 1200 North State St., Suite 3300 Los Angeles, CA 90033, USA; ^9^Department of Internal Medicine, Semnan University of Medical Sciences, Semnan, Iran; ^10^Department of Molecular and Cellular Sciences, Faculty of Advance Sciences and Technology, Pharmaceutical Sciences Branch, Islamic Azad University, Tehran, Iran; ^11^Department of Obstetrics and Gynecology, Shahid Beheshti University of Medical Sciences, Tehran, Iran; ^12^Department of Neurosurgery, Shiraz University of Medical Sciences, Shiraz, Iran; ^13^Department of Emergency Medicine, Loghman Hakim Hospital, Shahid Beheshti University of Medical Sciences, Tehran, Iran

## Abstract

The prefrontal cortex is the largest lobe of the brain and is consequently involved in stroke. There is no comprehensive practical pharmacological strategy for ameliorating prefrontal cortex injury induced by cerebral ischemia. Therefore, we studied the neuroprotective properties of verapamil (Ver) on mitochondrial dysfunction and morphological features of apoptosis in transient global ischemia/reperfusion (I/R). Ninety-six Wistar rats were allocated into four groups: control, I/R, I/R+Ver (10 mg/kg twice 1 hour prior to ischemia and 1 hour after reperfusion phase), and I/R+NaCl (vehicle). Animals were sacrificed, and mitochondrial dysfunction parameters (i.e., mitochondrial swelling, mitochondrial membrane potential, ATP concentration, ROS production, and cytochrome c release), antioxidant defense (i.e., superoxide dismutase, malondialdehyde, glutathione peroxidase, catalase, and caspase-3 activation), and morphological features of apoptosis were determined. The results showed that mitochondrial damage, impairment of antioxidant defense system, and apoptosis were significantly more prevalent in the I/R group in comparison with the other groups. Ver decreased mitochondrial damage by reducing oxidative stress, augmented the activity of antioxidant enzymes in the brain, and decreased apoptosis in the I/R neurons. The current study confirmed the role of oxidative stress and mitochondrial dysfunction in I/R progression and indicated the possible antioxidative mechanism of the neuroprotective activities of Ver.

## 1. Introduction

Stroke, as a leading cause of mortality, may contribute to major long-term morbidities [1]. One-third of strokes is reported to be fatal, and the rate and age of disease are strongly correlated with the country's economic status [2]. Ischemic stroke or cerebral ischemia is the most prevalent type of stroke (80%) and is a result of complete or partial interruption of blood flow through thrombus or embolism blockage in the cerebral arteries [3]. The brain consumes 20% of oxygen and glucose intake as its main energy substrate [4]. Therefore, it is very susceptible to disruption of metabolism and loss of aerobic glycolysis, resulting in cell death and possibly permanent neurological deficit [5]. The largest part of the brain, i.e., the frontal lobe, is affected more than other areas of the brain during stroke.

Currently, intravenous administration of thrombolytic agents (e.g., tissue plasminogen activator) and thrombectomy of the involved cerebral artery are considered as the main therapeutic interventions for acute ischemic stroke. However, these interventions are limited to a small percentage of patients due to their narrow therapeutic time window and complications such as hemorrhagic transformation [6].

Despite the importance of reperfusion after an acute cerebral infarction for prompt restoration of normal function, generation of reactive oxygen species (ROS) occurs at the onset, leading to oxidation of macromolecules, lipids, proteins, and DNA [7]. The high content of polyunsaturated fatty acids (PUFA) in neural cell membranes, low levels of endogenous ROS scavenging enzymes and antioxidants, and high aerobic metabolism render the brain very vulnerable to ROS damage [8].

The basal ROS levels are predominantly produced due to mitochondrial energy metabolism under physiological conditions and participate in vital processes [8], yet maintained in balance by antioxidant enzymes and antioxidants, which are interrupted after ischemia and reperfusion [9]. Therefore, oxidative stress occurs, which refers to the imbalance between the production of ROS (e.g., hydroxyl radicals, superoxide anions, peroxynitrite radicals, and hydrogen peroxides) and endogenous antioxidant enzymes (e.g., glutathione peroxidase [GPx], catalase [CAT], and superoxide dismutase [SOD]) or low-molecular-weight antioxidants, including reduced glutathione (GSH) [8]. ROS is found responsible for both necrotic and apoptotic neuronal cell deaths. Lipid peroxidation by ROS disintegrates membrane fluidity and causes cell injury. Also, ROS disturbs mitochondrial function, resulting in cell energy impairment, initiation of apoptosis cascade, and subsequent cell death [4, 10].

Considering the well-established role of ROS in stroke neuronal injury, efforts have been made to propose new neuroprotective agents and antioxidants in recent years [11]. Verapamil, a phenylalkylamine-class and an L-type voltage-gated calcium channel blocker, is routinely administered for the management of hypertension and angina pectoris. Calcium antagonists are known to be lipophilic and possess antiperoxidative properties against lipid peroxidation, probably due to having aromatic resonance rings, which can stabilize free radicals (in particular, verapamil) by having higher lipophilicity and concentration [12]. Verapamil also has been shown to decrease lipid peroxidation and enhance antioxidant enzyme activity; it also possesses protective properties against ROS in diabetic nephropathy [13]. Therefore, we investigated mitochondrial changes, as well as morphological features of apoptosis, in a transient global ischemia/reperfusion (I/R) rat model.

## 2. Methods

### 2.1. Chemicals and Regents

The SYBR Premix Ex Taq™ II kit and cDNA synthesis kit were obtained from Takara (Japan). The TRIzol reagent and Caspase-3 Colorimetric Assay Kit were obtained from Invitrogen (USA) and Abcam (USA), respectively. Fluorescein *In Situ* Cell Death Detection Kit and DNase grade I solution were purchased from Roche Molecular Biochemicals (Germany). The R&D Systems (USA) and Vector (USA), respectively, provided the Quantikine Cytochrome c ELISA Assay and Vectashield Mounting Medium. Sigma-Aldrich Co. (Germany) supplied the rest of the commercial-grade materials.

### 2.2. Animals

A total of 96 male Wistar rats weighing 250–300 g were obtained from the Pharmacology Department of Tehran University of Medical Sciences. The rats were kept in the standard cage and under a 12-hour light/dark cycle and were allowed free access to food and water *ad libitum*. They were also kept in the animal house for a week prior to the experiments. All the experiments adhered to the ARRIVE ethical guidelines [14]. The Ethics Committee of the Tehran Medical Sciences Branch, Islamic Azad University, also approved the experiments. Also, the Guide for the Care and Use of Laboratory Animals (edition 1985, NIH Publication No. 86-23) was followed during the experiments.

### 2.3. Surgical Procedure

The four-vessel occlusion model was applied to induce transient global forebrain ischemia based on the method proposed by Pulsinelli and Buchan [15]. In brief, the animals underwent general anesthesia via intraperitoneal (i.p.) pentobarbital sodium (40 mg/kg). The vertebral arteries were electrocoagulated from the alar foramen of the first cervical vertebra and subjected to bilateral common carotid artery occlusion after 24 hours. Rats were placed on the back, and a 2 cm ventral neck incision was made; both common carotid arteries were separated carefully from the vagus nerve and occluded by using nontraumatic clamps. After 20 min of ischemia, the clamps were removed. The change of color from pale white to reddish confirmed the reperfusion of blood flows into the common carotid artery. Rats were returned to their cage after the surgery and kept separately for 96 hours.

### 2.4. Drug Administration and Experimental Groups

The rats were randomly divided into four groups (24 rats per group):

- *Control group*: rats received no drug or surgical procedure
- *I/R*: rats were subjected to transient global cerebral I/R
- *I/R+Ver*: rats were subjected to I/R and treated with verapamil at an effective dose of 10 mg/kg/i.p. twice: 1 h prior to ischemia and 1 h after the reperfusion phase [16, 17]
- *I/R+NaCl*: rats were subjected to I/R and received an equal volume of vehicle (0.09% NaCl, i.p.) at the same time period

### 2.5. Brain Sample Preparation

Five minutes before perfusion, anesthesia was induced with xylazine and ketamine (i.p.; 10 and 50 mg/kg, respectively). The rats (6 rats per group) were perfused with PBS and then 4% paraformaldehyde (pH = 7.4) for the histopathological analysis. After the brains were postfixed at 4°C for three days, they were paraffin-embedded, cut into 3 *μ*m TUNEL and 10 *μ*m Nissl sections (0.2 to 1.2 mm posterior to the bregma), and mounted on 2% 3-aminopropyltriethoxysilane-coated glass slides. The rats (*n* = 18 per group) were perfused transcardially by normal saline (pH = 7.4); following that; they were decapitated for molecular and biochemical analyses. After removing the cerebellum and olfactory bulb, liquid nitrogen was used to snap-freeze the brain hemispheres, then they were stored at −80°C.

### 2.6. Isolation of Prefrontal Cortex Mitochondria

Differential centrifugation was used to prepare the mitochondria from the prefrontal cortex. After removing the tissues, they were minced in a cold mannitol solution (75 mM sucrose, 0.2 mM EDTA, and 0.225 M D-mannitol) by a small scissor. Using a Teflon pestle, the tissues were gently homogenized in a glass homogenizer, followed by centrifugation at 1000 × g for 10 minutes (4°C) to eliminate the nonsubcellular debris, nuclei, and unbroken cells.

Centrifugation was performed for 10 minutes at 10,000 × g. After resuspending the mitochondrial fraction (dark packed lower layer) in mannitol solution, it was recentrifuged twice for 10 minutes at 10,000 × g. Before the assay, the mitochondrial sediments were suspended at 4°C in a solution of 2.0 mM MgCl_2_, 20 mM KCl, 0.25 M sucrose, 1.0 mM Na_2_HPO_4_, and 0.05 M Tris-HCl buffer (pH, 7.4). For determining the oxidative stress parameters, we used the suspension aliquots. All the tests were repeated three times.

#### 2.6.1. The Protein Concentration of Mitochondria of the Prefrontal Cortex

For determining the protein concentrations, the Bradford assay was applied [18]. The level of succinate dehydrogenase was measured to confirm the isolation of mitochondria [19]. In each experiment, the mitochondria were freshly prepared and used after 4 hours of isolation. All the steps were followed strictly on ice to ensure the isolation of preparations with high quality.

#### 2.6.2. Mitochondrial ROS Measurement

For this purpose, the mitochondria were added to a respiration buffer of 10 mM Tris, 50 *μ*M EGTA, 0.32 mM sucrose, 0.1 mM KH_2_PO_4_, 20 mM MOPS, 5 mM sodium succinate, and 0.5 mM MgCl_2_ [20]. After collecting the samples, DCFH-DA (final concentration, 10 *μ*M) was added to the mitochondria, and incubation was performed for 15 minutes. A fluorescence spectrophotometer (Shimadzu RF-5000U) was used to measure the fluorescence intensity of DCF at emission and excitation wavelengths of, respectively, 527 and 488 nm.

#### 2.6.3. Determined GSH Content as the Antioxidative Content

The mitochondrial fractions were added to DTNB 0.04% (total volume, 3.0 mL; pH, 7.4) and phosphate buffer (0.1 mol/L). Then, a UV-1601 PC spectrophotometer (Shimadzu, Japan) was used to read the yellow color produced at 412 nm. The level of GSH was presented as *μ*g/mg protein.

#### 2.6.4. Mitochondrial Membrane Potential (MMP) Assay

For MMP analysis, the mitochondrial uptake of rhodamine123, a fluorescent cationic dye, was determined. Then, 500 *μ*g protein/mL of mitochondrial suspensions was incubated with rhodamine123 (10 RM) in the MMP buffer (5 mM KH_2_PO_4_, 220 mM sucrose, 10 mM KCl, 68 mM D-mannitol, 2 mM MgCl_2_, 50 *μ*M EGTA, 2 *μ*M rotenone, 10 mM HEPES, and 5 mM sodium succinate).

A fluorescence spectrophotometer (Shimadzu RF-5000U) was used to measure fluorescence at emission and excitation wavelengths of 535 and 490 nm, respectively [21]. The mitochondrial uptake of rhodamine123 was determined as the difference in rhodamine123 fluorescence between the treated and control mitochondria. Values are presented as the MMP collapse percentage (% *ΔΨ*m) in the treated groups.

#### 2.6.5. Determination of Mitochondrial Swelling

A swelling buffer, consisting of 70 mM sucrose, 230 mM mannitol, 3 mM HEPES, 2 mM Tris-phosphate, 1 RM rotenone, and 5 mM succinate, was used to suspend the isolated mitochondria [22]. Using an ELISA reader (Tecan, Rainbow Thermo, Austria), absorbance was determined at 540 nm within 10-minute intervals. Reduction in the level of absorbance represents the increased level of mitochondrial swelling.

#### 2.6.6. Cytochrome c Release Assay

The Quantikine Rat/Mouse Cytochrome c ELISA Kit was used to determine the cytochrome c level. After precoating the microplate with cytochrome c monoclonal antibody, 75 *μ*L of the conjugate (i.e., cytochrome c monoclonal antibody conjugated to horseradish peroxidase), as well as 50 *μ*L of the test and control samples, was added to each well. Following that, from every supernatant fraction, 1 *μ*g of protein was added to the wells. In addition, the standard, test, and control samples were added to the wells of the microplate. After 2 hours of incubation, 100 *μ*L of substrate solution was added to each well, followed by reincubation for 30 minutes. The microplate spectrophotometer was also used to measure optical density (450 nm) after adding a stop solution (100 *μ*L) to each well.

#### 2.6.7. Assay Mitochondrial ATP Content of Prefrontal Cortex

The luciferin/luciferase enzyme system was used to determine the level of ATP [23]. In addition, a Sirius tube luminometer (Berthold Detection System, Germany) was used to measure the bioluminescence intensity. The ATP level was expressed as *μ*g/mg protein.

### 2.7. Determination of Oxidative and Antioxidative Content of Prefrontal Cortex

After 96 hours of I/R, the rats under anesthesia (*n* = 6 per group) were decapitated. Following the immediate removal of the brain, the prefrontal cortex was removed and weighed. Ice-cold triple distilled water (2 mL) was used to homogenize the tissues, followed by sonication for 15 seconds. After centrifugation of the homogenates for 2 minutes at 10,000 × g, enzyme estimation was carried out using the supernatants. Also, the method proposed by Lowry et al. was applied for protein estimations [24].

#### 2.7.1. Measurement of Malondialdehyde (MDA) Level

For evaluating the MDA level, the method proposed by Esterbauer and Zollern, based on thiobarbituric acid reactive substances (TBARS), was applied [25]. The values were presented as nmol per mg of wet tissue.

#### 2.7.2. Determination of SOD Activity

The method introduced by Durak et al., which is dependent on the inhibition of nitroblue tetrazolium (NBT) reduction, was applied to determine the SOD activity [26]. For this purpose, after adding the mixture of ethanol and chloroform (1.0 mL; 5 : 3, *v*/*v*), the ethanol phase of the supernatant was added to the sample and centrifuged. The amount of enzyme, producing 50% NBT reduction inhibition, was defined as 1 unit of SOD. The level of SOD was presented as U/mg protein.

#### 2.7.3. CAT Assay

Using Claiborne's method, CAT activity was measured in tissues [27]. For this purpose, a spectrophotometer (UV-1700, Shimadzu, Japan) was employed at 240 nm to determine the disappearance of H_2_O_2_. The CAT activity was presented as U/mg of protein, where 1 unit represents the amount of protein needed for degradation of 1 *μ*mol of H_2_O_2_ per minute.

#### 2.7.4. Measurements of GPx Level

The method introduced by Rotruck et al. was employed to estimate the level of GPx [28]. In brief, incubation of the reaction mixture (0.4 mL of Tris-HCl buffer, 0.2 mL of GSH, 0.1 mL of water, 0.1 mL of sodium azide, 0.1 mL of H_2_O_2_, and 0.1 mL of homogenate) was performed for 15 minutes at 37°C, then trichloroacetic acid (TCA; 0.5 mL) was added and centrifuged. Following that, the supernatant (0.5 mL) was collected, and Ellman's reagent (0.5 mL) and Na_2_HPO_4.2_H_2_O (2 mL) were added. GPx activity was presented as *μ*mol/min/mg protein, and absorbance was read at 420 nm.

### 2.8. RNA Extraction and Real-Time PCR Assay for Tp53, Caspase-8, Bax, Cytochrome c, and Bcl-2 Genes

Based on the standards, a TRIzol reagent was used to extract total RNA. Then, using DNase I, the RNA samples were treated. Also, a cDNA synthesis kit was used to synthesize total cDNA, as outlined by the kit instructions. For amplification of cDNA from Tp53, caspase-8, Bax, cytochrome c, Bcl-2, and GAPDH (an internal control for normalization), the primers were specifically designed. Table S1 presents specific primers and target genes.

The 7500 Fast Real-Time System (Applied Biosystems, USA), along with the SYBR Premix Ex Taq™ II kit, was employed to perform cDNA amplification. The PCR reactions included holding (95°C for 30 seconds) and cycling (denaturation for 5 seconds at 95°C, followed by annealing/amplification for 30 seconds at 60°C) stages; the total number of cycles was 40. The 2-*ΔΔ*Ct method was applied for the measurement of relative gene expression. Changes in the target gene, cDNA, with respect to the internal control (GAPDH) were measured using the following formula:

(1) $$\begin{array}{c} \text{Change}=2‐\Delta \Delta \text{Ct}, \end{array}$$

where

(2) $$\begin{array}{c} \Delta \Delta \text{Ct}=\left(\text{Ct target gene}-\text{Ct GAPDH}\right)-\left(\text{Ct control}-\text{Ct GAPDH}\right). \end{array}$$

### 2.9. Measurement of Caspase-3 Activity of the Prefrontal Cortex

In accordance with the kit instructions, the caspase-3 colorimetric assay was used to determine caspase-3 activity. After homogenization and centrifugation (temperature, 4°C; duration, 1 minute; speed, 10,000 g) of the prefrontal cortex tissue blocks, the supernatant was extracted and assessed in terms of the extracted protein samples (200 *μ*g), which were incubated with Ac-DEVD-caspase-3-like inhibitor and reaction buffer and at 37°C for 60 minutes. A spectrophotometer was used to measure the enzyme-catalyzed release of paranitroaniline (p-NA) at 400-450 nm. Following incubation with the substrate, optical density was determined. The changes in optical density indicated the increase in caspase-3 activity.

### 2.10. Histopathological Assessments

#### 2.10.1. TUNEL Staining

Neuronal cell death was determined in tissue sections using the TUNEL method via the *In Situ* Cell Death Detection Kit. Briefly, after drying the slides for 30 minutes, they were fixed at room temperature in 10% formalin solution. The sections, after being washed in PBS, were incubated in ethanol : acetic acid solution (3 : 1) and washed again with PBS. For permeabilization, incubation was carried out with 3% Triton X-100 solution at room temperature for 1 hour.

Afterward, the TdT enzyme was used to incubate the slides in fluorescein dUTP (a reaction buffer) at 37°C for 90 minutes. For the negative control, only the reaction buffer was used without any TdT enzymes, while for the positive controls, DNase grade I solution (500 U/mL) was used for digestion. The Vectashield® mounting medium containing DAPI was used to cover the slices and to preserve the cells for comparison. After staining, the samples were immediately examined by a fluorescence microscope (Axioskop 40, Zeiss, Germany) at 520 and 460 nm for TUNEL fluorescence and DAPI, respectively.

#### 2.10.2. Nissl Staining

According to the protocols, 10 *μ*m paraffin sections were dewaxed, rehydrated, and stained using 5% cresyl violet as previously described [29]. The sections were dehydrated in increasing ethanol concentrations and then cleared in xylene, following rinsing with double distilled water. They were observed under a light microscope after being mounted with a paramount coverslip. The normal neurons consisted of cells containing the Nissl substance in the cytoplasm, prominent nucleoli, and loose chromatin, while damaged neurons were characterized by cavitation around the nucleus, loss of Nissl substance, and pyknotic nuclei.

### 2.11. Cell Counting and Histopathological Image Analysis

Five visual fields (0.25 mm × 0.25 mm) of the prefrontal cortex were imaged from each section. The Nissl and TUNEL-positive cells were counted in a high-power field (×400) by the Image-Pro Plus (Leica DMLB, Germany). Also, the cell count was averaged (number/mm^2^) in the same group of animals. A pathologist, blinded to the treatment, performed the microscopic examinations.

### 2.12. Statistical Analysis

Values are presented as mean ± SD. For evaluating the intergroup differences, ANOVA and Tukey's posttest were applied. GraphPad Prism v. 5.0 (USA) was used in this study, and the significant difference was set at 0.05.

## 3. Results

### 3.1. Assessment of the Mitochondrial Toxicity

#### 3.1.1. Verapamil Reduced the ROS Formation in Isolated Prefrontal Cortex Mitochondria

**Figure 1:**
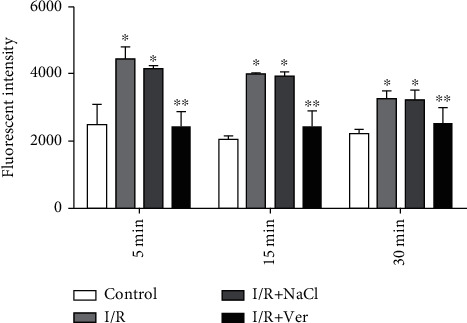
ROS formation in isolated prefrontal cortex mitochondria. The data are presented as mean ± SD (*n* = 6). ^∗^*P* < 0.001 vs. the control group, ^∗∗^*P* < 0.001 vs. I/R+NaCl and I/R groups.

In contrast with the control group, in the I/R and I/R±NaCl groups, ROS formations were considerably (*P* < 0.001) increased in isolated prefrontal cortex mitochondria (Figure 1). However, the treatment of rats with verapamil significantly (*P* < 0.001) reduced ROS formation. The ROS formation was measured in the time intervals (5, 15, and 30 minutes) following isolation of rat prefrontal cortex mitochondria in all groups.

### 3.2. Determining the Antioxidative Activity in the Mitochondria Isolated from the Prefrontal Cortex

#### 3.2.1. Verapamil Administration Could Increase the GHS Content of Prefrontal Cortex of Rats following I/R Damage

The GHS content after induction of I/R in the rats significantly decreased compared to control rats (Fig. S1). In contrast, GSH content in the I/R±Ver group was markedly higher than that of all groups (*P* < 0.001).

#### 3.2.2. Effect of Verapamil on the MMP to Reverse the Oxidative Stress Induced by I/R Damage

**Figure 2:**
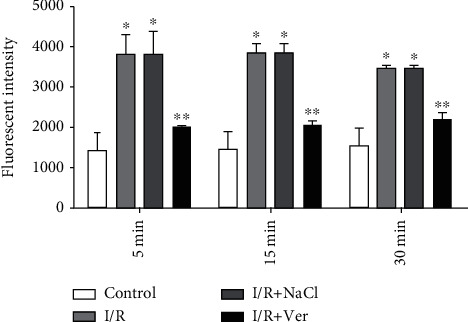
MMP decrease in the prefrontal cortex mitochondria isolated from rats. The data are presented as mean ± SD (*n* = 6). ^∗^*P* < 0.001 vs. control group, ^∗∗^*P* < 0.01 vs. I/R+NaCl and I/R groups.

The MMP decreased in the mitochondria isolated from the prefrontal cortex of I/R and I/R+NaCl groups compared with the control group (Figure 2, *P* < 0.001). As shown in Figure 2, the treatment of rats with verapamil could significantly inhibit the decrease of MMP in comparison with the I/R and I/R+NaCl groups (*P* < 0.01).

#### 3.2.3. Verapamil Ameliorated Mitochondrial Swelling of the Prefrontal Cortex

**Figure 3:**
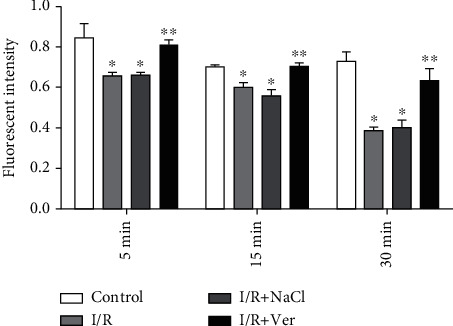
Progressive mitochondrial swelling in the prefrontal cortex mitochondria isolated from experimental rats. The data are presented as mean ± SD (*n* = 6). ^∗^*P* < 0.001 compared with the control group, ^∗∗^*P* < 0.01 vs. I/R+NaCl and I/R groups.

Figure 3 indicates that the mitochondrial swelling in the I/R group was significantly (*P* < 0.001) higher than that of the control group. However, administered verapamil decreased substantially the mitochondrial swelling compared with I/R+NaCl and I/R groups.

#### 3.2.4. Verapamil Administration Increased the Mitochondrial ATP Level of Prefrontal Cortex following I/R Damage

Transient global cerebral induced I/R indicate a dramatic reduction (*P* < 0.001) in the ATP level of mitochondria isolated from the prefrontal cortex when compared to the control group (Fig. S2). Treatment with verapamil significantly (*P* < 0.001) increased the ATP level in the prefrontal cortex of the I/R+Ver group when compared to other groups.

#### 3.2.5. Verapamil Decreases Cytochrome c Release in Isolated Prefrontal Cortex Mitochondria

Cytochrome c release, the endpoint of mitochondrial toxicity, was significantly increased in the I/R group compared with the control group (*P* < 0.001, Fig. S3). However, cytochrome c release in the I/R±Ver group was markedly lower than that in all the groups except the control group (*P* < 0.001).

### 3.3. Biochemical Estimation

#### 3.3.1. Effect of Verapamil on Prefrontal Cortex MDA Content

The level of brain MDA in the I/R group was significantly (*P* = 0.033) higher than that in the control group. Verapamil administration could dramatically inhibit the increase of MDA in comparison with the I/R and I/R+NaCl groups (*P* = 0.017 and *P* = 0.014, respectively, Table 1).

#### 3.3.2. Verapamil Improved Antioxidative Activity via Increased SOD Content of Prefrontal Cortex

As shown in Table 1, the activity of SOD in the I/R rats was significantly (*P* = 0.008) lower than that in the control rats. The SOD content of the brain of the I/R+Ver group was very low, similar to the level found in the control group. Verapamil-treated rats have a significant (*P* = 0.028) increase of SOD activity in comparison with the I/R group.

#### 3.3.3. Verapamil Treatment Enhanced the CAT Content of Prefrontal Cortex

Regarding Table 1, the activity of CAT in the I/R rats was significantly (*P* = 0.006) lower than that in the control rats. The administered verapamil induced a significant increase in the CAT activity in the prefrontal cortex of the I/R+Ver group compared with I/R+NaCl and I/R groups (*P* = 0.018 and *P* = 0.036, respectively).

#### 3.3.4. Verapamil Increments the GPx Content of Prefrontal Cortex following I/R

Transient global cerebral induced I/R showed a significant (*P* = 0.037) decrease in the prefrontal cortex GPx level in comparison to the control group (Table 1). Administration of verapamil markedly (*P* = 0.005) induced the increase in the level of GPx in the prefrontal cortex of the I/R+Ver group in comparison with the control group (Table 1).

### 3.4. Verapamil Enhanced the Expression of Antiapoptotic mRNAs and Reduced the Proapoptotic mRNAs in the Prefrontal Cortex following I/R

**Figure 4:**
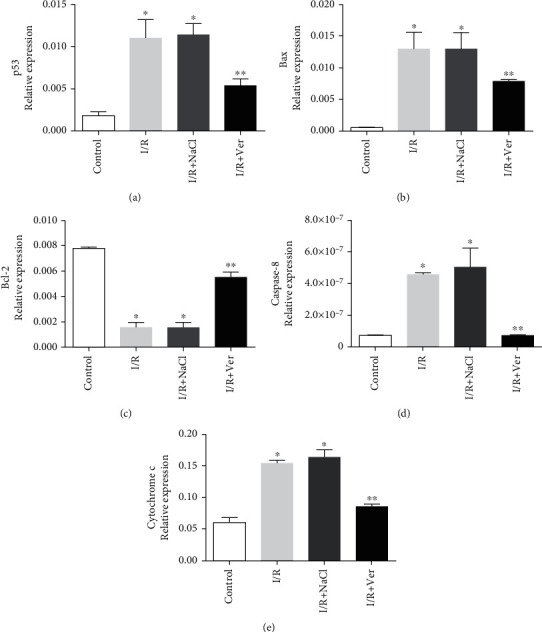
Quantitative real-time polymerase chain reaction (PCR) analysis of relative expression mRNAs in the prefrontal cortex. The p53 (a), Bax (b), Bcl-2(c), caspase-8 (d), and cytochrome c (e). Verapamil treatment could significantly reduce the expression of proapoptotic mRNAs (Bax, p53, cytochrome c, and caspase-8) and improved antiapoptotic (Bcl-2) mRNAs. Data are presented as means ± SD of the normalized PCR product concentrations for each dilution step. ^∗^*P* < 0.01 compared with the control group, ^∗∗^*P* < 0.01 vs. I/R+NaCl and I/R groups.

Relative expression of Bax, p53, cytochrome c, and caspase-8 mRNAs in the prefrontal cortex of I/R and I/R+NaCl groups was higher than those of the control group, while relative expression of Bcl-2 mRNA was significantly lower than those of the control group (Figures 4(a)–4(e); *P* < 0.01). As showed in Figure 4, the treatment of rats with verapamil effectively decreased the relative expression of proapoptotic mRNAs, such as Bax, p53, cytochrome c, and caspase-8 (Figures 4(a), 4(b), 4(d), and 4(e); *P* < 0.01), and increased the relative expression Bcl-2 in the I/R+Ver group compared with I/R and I/R+NaCl groups (Figures 4(c) and 4(e); *P* < 0.01).

### 3.5. Administration of Verapamil Could Reduce the Caspase-3 Activity in the Prefrontal Cortex

We evaluated caspase-3 activity by spectrophotometer. The higher absorbance value indicated the higher caspase-3 activity and, as a result, higher incidence of apoptosis. Caspase-3 activity for the control group was lower than that for the I/R and I/R+NaCl groups (0.02 ± 0.01 vs. 1 ± 0.01 and 0.82 ± 0.09, *P* < 0.001, respectively). Treatment rats with verapamil significantly reduce the caspase-3 activity in the prefrontal cortex (*P* < 0.005, Fig. S4).

### 3.6. The Treatment with Verapamil Reduced Neurodegeneration in the Prefrontal Cortex of Transient Global Cerebral I/R-Induced Rats

**Figure 5:**
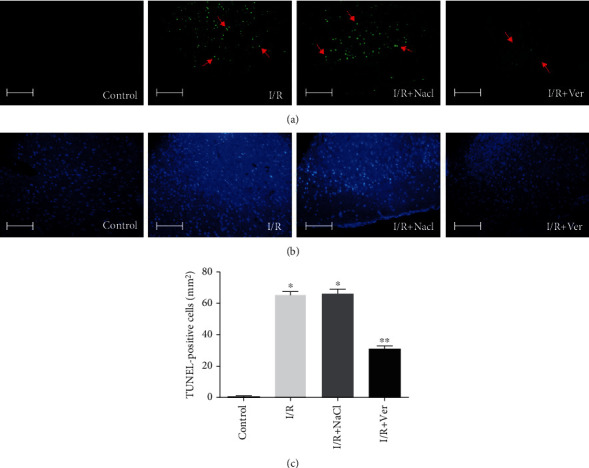
Effects of verapamil on transient global I/R-induced apoptotic neurodegeneration. Representative photomicrographs of TUNEL staining and cell counting. (a) Representative images of the TUNEL-positive cell (arrows) were obtained from sections prepared from the animals in control, I/R, I/R+NaCl, and I/R+Ver groups. (b) DAPI nuclear staining was indicated overall in the cellular morphology of the TUNEL sections. (c) Treatment rats with verapamil significantly reduced the TUNEL-positive cell compared to I/R and I/R+NaCl groups. Data are represented as mean ± SD. Scale bars 100 *μ*m, magnification ×400. ^∗^*P* < 0.005 vs. control group, ^∗∗^*P* < 0.005 vs. I/R and I/R+NaCl groups.

To determine the apoptotic cells in the prefrontal cortex of rats, the TUNEL assay was performed. The TUNEL-positive cells were detected in the injured prefrontal cortex. TUNEL-positive cells were numerous in the I/R and I/R+NaCl groups. Accordingly, it showed chromatin condensation and numerous dense masses of membrane-bound apoptotic bodies (Figures 5(a) and 5(b)). Meanwhile, it indicates a negative finding in the control group. However, verapamil administration markedly reduced the number of TUNEL-positive cells (*P* < 0.005, Figure 5(c)).

### 3.7. Verapamil Could Augment the Neuron Survival of Prefrontal Cortex following I/R

**Figure 6:**
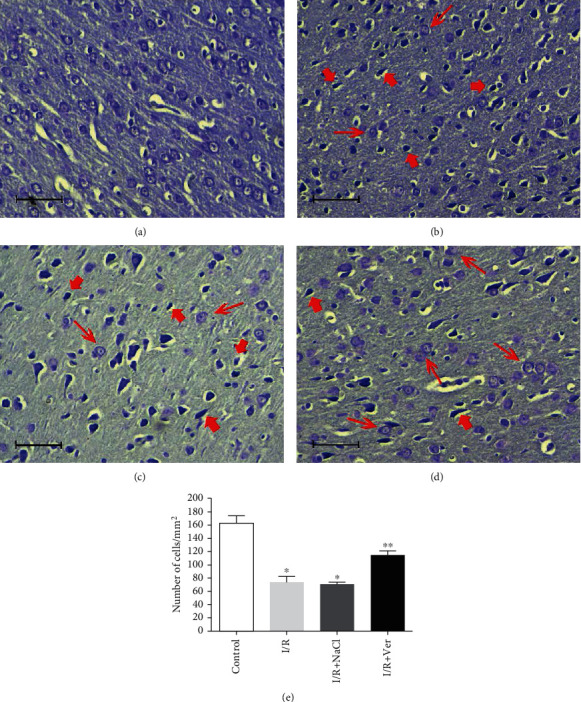
Nissl staining of rats' prefrontal cortex following transient global I/R and cell counting. The prefrontal cortex of control animals (a) did not contain any damaged neurons. However, the cortex of rats in the I/R (b) and I/R+NaCl (c) groups was characterized by fewer Nissl stain neurons (thin arrows) than that in the I/R+Ver group (d) and markedly contained shrunken, intensely stained, and dystrophic neurons (thick arrows). As shown in the graph (e), treatment rats with verapamil significantly reduced neuronal cell loss in the prefrontal cortex. Data are represented as mean ± SD. Scale bars 100 *μ*m, magnification ×400. ^∗^*P* < 0.001 vs. control group, ^∗∗^*P* < 0.001 vs. I/R+NaCl and I/R groups.

Figure 6 shows representative samples of Nissl staining from the prefrontal cortex of rats 96 h after I/R. In the I/R and I/R+NaCl groups, numerous damaged neurons with shrunken cytoplasm and pyknotic nuclei were observed (Figures 6(b) and 6(c)), while no morphological changes were seen in the control group (Figure 6(a)). Verapamil treatment preserved the integrity of neurons within the prefrontal cortex (Figure 6(d)). Also, the number of survived neurons in the I/R group was significantly lower than that in the control group (73.4 ± 8.87 vs. 162.8 ± 11.58, *P* < 0.001, respectively). Treatment with verapamil significantly prevented the neuron loss compared with that in the I/R and I/R+NaCl groups (114.6 ± 6.5 vs. 73.4 ± 8.87 and 71.2 ± 2.1, *P* < 0.001, respectively; Figure 6(e)).

## 4. Discussion

This study was conducted on 96 male Wister rats that underwent transient global forebrain ischemia using the four-vessel occlusion method. Rats were divided into four equal groups: control, I/R, I/R with NaCl, and I/R treated with verapamil.

Verapamil recovers microvascular dysfunction by preventing vascular spasm and reducing vessel dysfunction [30]. Calcium channel blockers have been used for hypertension, traumatic brain injury, intracerebral hemorrhage, etc. [31]. Inflammation process and microglia cell activities have a double-edged sword in stroke [32].

Verapamil-treated rats had lower levels of ROS compared to the I/R and I/R+NaCl groups in our study. The ROS components could cause neurological cell injury [33, 34]. We found out that verapamil significantly reduced ROS formation after 20 minutes of the stroke. Thangapazham et al. showed similar findings in their experiments [35]. The nuclear factor *κ*B (NF-*κ*B) signaling pathway is the backbone of inflammation in human cells [36–38]. Yan and Li showed that NF-*κ*B had been changed by verapamil [37].

Activated T cells and macrophage overexpression of CD40 and CD40L during inflammation and activated smooth muscles and endothelial cells can lead to the production of matrix metalloproteinases [39]. According to our results, verapamil may reduce the matrix metalloproteinases and have protective effects on local inflammation and cell injury after stroke. Neural cell injuries after stroke can make local or semiglobal cell necrosis and may start harmful positive feedback developing into brain inflammation, cytochrome C release, and mitochondrial damage [40].

MDA is a poisonous byproduct of LPO and a reliable indicator of oxidative stress [41]. The end product of lipid peroxidation is MDA. The MDA was significantly lower in the verapamil group compared to the I/R groups. This can be due to both reduced production and increased scavenging capacity of ROS.

The antioxidant defense system tries to combat ROS excess. The SOD, an antioxidative metalloenzyme, catalyzes the dismutation of superoxide to hydrogen peroxide, which is subsequently scavenged by peroxisomal catalase and GPx to water and molecular oxygen. Using hydrogen peroxidase, GPx oxidizes GSH, one of the primary endogenous antioxidants [42].

Eventually, this complex cascade of interactions intercepts ROS-induced cellular damage [43]. In line with the neuroprotective effect of verapamil in this study, all measured antioxidants, SOD, CAT, GPx, and GSH, were strongly boosted by verapamil, with higher levels compared to all other groups, which supports the beneficial role of verapamil in aiding the antioxidant capacity of neurons. Previous studies have shown that the endogenous antioxidant enzymes such as SOD, GPx, and CAT, along with low-molecular-weight antioxidants, like GSH [3, 44], are protective against stress oxidation events [45].

Mitochondria are responsible for energy metabolism via ATP production and calcium homeostasis, in addition to being a significant source of ROS during reperfusion, leading to necrotic and intrinsic- and extrinsic-mediated apoptotic cell deaths. Excessive production of ROS companies with calcium loading leads to failure of the permeability barrier of the inner mitochondrial membrane, allowing for the entrance of solutes and water, which result in swelling of mitochondria. Our results show that mitochondrial swelling in the verapamil-treated group was shown to be greater than that in the controls and milder than that in the I/R groups. This swelling in turn ruptures the outer membrane and results in releases of apoptotic-promoting proteins like cytochrome c, apoptosis-inducing factor, and caspases into the cytosol to initiate the intrinsic pathway of apoptosis [43, 44]. Caspases are cysteine-dependent proteases known for their crucial role in the mediation of cell apoptosis [44].

Caspase-3, as the most abundant caspase in the brain, can be activated by both intrinsic and extrinsic pathways. It is upregulated after ischemic stroke and stimulates caspase-activated DNAse, thus causing DNA fragmentation, while its inhibition ameliorates ischemic cell injury [1, 5].

As our results showed, the rise in both proapoptotic factors, caspase-3, and cytochrome c was prevented to some extent in the verapamil group. This finding confirms that verapamil ameliorated cell injury by indirect inhibition of apoptotic pathways. Apoptosis manifests morphological characteristics, including DNA fragmentation, the formation of apoptotic bodies, and shrinkage of the cell [10]. Our results also confirmed the neuroprotective effect of verapamil on neuronal cell death through TUNEL and Nissl staining.

del Zoppo et al. demonstrated that a mild inflammation has occurred after ischemic stroke, with cytokines such as TNF-*α* and IL-1*β* increasing. So, apoptosis is stimulated around the ischemic area [46]. In our study, the activity of caspase-3 in the verapamil group was lower than others, so verapamil could have helped reduce the inflammation effects on cell apoptosis. Indeed, microglial cells may secrete proinflammatory or anti-inflammatory cytokines [47], so if the balance of this cytokine is thrown, it could have harmful positive feedback on cell apoptosis after stroke [48].

As the ATP level decreases during ischemia, Na/K ATPase and Ca/Mg ATPase, the critical membrane proteins, which maintain membrane potential and ion concentration, are therefore inhibited and cannot transport ions across the membrane. The rapid depletion of ATP is thus very detrimental to cells. The activity of Na^+^/K^+^-ATPase is another indicator of oxidative stress [49]. Also, lipid peroxidation inhibits Na^+^/K^+^-ATPase, which impairs membrane permeability and brings about electrochemical gradient decline and osmotic damage [7, 49], and eventually cell death. Consistent with other findings, verapamil was shown to have a protective effect on maintaining MMP.

Interleukin-1*β*, Bcl-2-associated X protein, and p53 gene overexpression are essential in animal cell apoptosis, and determination of the Bax/Bcl-2 ratio is a good indicator of cell apoptosis status [50]. The Bcl-2 family proteins are key regulating factors in intrinsic apoptosis pathways. They serve as both anti- and proapoptotic mediators. The Bcl-2 is an inhibitory protein through the prevention of ROS generation and lipid peroxidation, whereas Bax is an inducing protein both belonging to this family [1, 7]. In verapamil-treated rats, Bcl-2 was expressed more than in I/R groups, contrary to Bax, which can be interpreted as the protective effect of verapamil on the regulation of the apoptotic pathway. It seems that verapamil could have a protective effect on acute cell stress and could regulate apoptosis, the same as other studies showed in the past [51, 52]. Scorrano et al. reported that Bax protein, Ca^2+^, works as a control point for apoptosis [52]. Verapamil is an efficient and specific calcium channel blocker, so it could be a good poetical neuroprotection during stroke and cell oxidation stress.

### 4.1. Limitations

The current study has some limitations. Regarding the previous evidence, the mitochondrial Ca^2+^ homeostasis plays a key role in aerobic metabolism and cell survival. However, as one of the main limitations of the current study, we only assessed the mitochondrial toxicity. Although the proper studies were performed to prove adequate evidence of the antiapoptotic effect of verapamil, some other tests (e.g., flow cytometry and Western blot) were not applied for verification of apoptosis, and this issue should be considered as another limitation.

## 5. Conclusion

In this study, various methods were utilized to assess the effect of verapamil on neuronal cell damage after I/R injury. Verapamil has protective effects in three steps, first in all antioxidation capacities, second in intrinsic and extrinsic apoptosis pathways, and third in the regulation of genes and inflammatory signaling pathways after stroke. The ROS production, scavengers, PCR, ATP production, mitochondrial membrane potential, and swelling, as well as cell death evaluation, revealed that verapamil could have a neuroprotective effect postacute ischemia.

## Figures and Tables

**Table 1:** The effect of verapamil on the brain biochemical enzyme content. Data are presented as mean ± SD.

Groups	MDA (nmol/mg of protein)	SOD (U/mg of protein)	CAT (U/mg of protein)	GPx (*μ*mol/mg of protein)
Control	56.91 ± 1.23	5.83 ± 0.16	6.05 ± 0.24	1.36 ± 0.11
I/R	121.08 ± 1.09^a^	8.08 ± 0.21^a^	7.25 ± 0.22^a^	0.45 ± 0.6^a^
I/R+NaCl	120.25 ± 1.21^a^	7.96 ± 0.13^a^	7.35 ± 0.34^a^	0.51 ± 0.07^a^
I/R+Ver	87.16 ± 0.94^a,b^	15.76 ± 0.41^a,b^	8.6 ± 0.14^a,b^	2.71 ± 0.19^a,b^

^a^ *P* < 0.05 vs. control group, ^b^*P* < 0.05 vs. I/R and I/R+NaCl groups.

## Data Availability

The data used to support the findings of this study are available from the corresponding author upon request.

## Abbreviations

t-PA: Tissue-plasminogen activator
ROS: Reactive oxygen species
PUFA: Polyunsaturated fatty acid
SOD: Superoxide dismutase
GPx: Glutathione peroxidase
CAT: Catalase
GSH: Glutathione
I/R: Ischemia/reperfusion
i.p.: Intraperitoneal
MMP: Mitochondrial membrane potential
MDA: Malondialdehyde
NBT: Nitroblue tetrazolium
PCR: Polymerase chain reaction
GAPDH: Glyceraldehyde 3-phosphate dehydrogenase
p-NA: p-Nitroaniline
TUNEL: TdT-mediated dUTP nick-end labeling
DAPI: 4′,6′-Diamino-2-phenylindole
NF-*κ*B: Nuclear factor *κ*B.

## Supplementary Materials

Supplementary Materials Table S1: details of primers (forward and reverse) for target genes used for RT-PCR analysis. Fig. S1: the GSH content in isolated prefrontal cortex mitochondria. Fig. S2: the ATP levels in the isolated prefrontal cortex mitochondria were determined using luciferin/luciferase enzyme system. Fig. S3: the cytochrome c release in the isolated prefrontal cortex mitochondria was measured by ELISA kit. Fig. S4: effect of the verapamil on the caspase-3 activity in the prefrontal cortex of rats.
