# Supplementary material for: Verapamil Inhibits Mitochondria-Induced Reactive Oxygen Species and Dependent Apoptosis Pathways in Cerebral Transient Global Ischemia/Reperfusion
Source: Oxid Med Cell Longev. 2020 Oct 17;2020:5872645. doi: 10.1155/2020/5872645 (PMC7591985; doi:10.1155/2020/5872645)

Supplementary Materials

Additional Supplementary Materials may be found in the online version of this article:

Table S1. Details of primers (forward & reverse) for target genes used for RT-PCR analysis.

**Gene**	**Primers sequence (5′-3′)**	**Product length**
Caspase 8	F: AGCAGCCTATGCCACCTAGT<br>R: GCTGTAACCTGTCGCCGAG	261 bp
Tp53	F: GGTACCGTATGAGCCACCTG<br>R: AACCTCAAAGCTGTCCCGTC	166 bp
Bax	F: CCAAGAAGCTGAGCGAGTGT<br>R: CCCAGTTGAAGTTGCCGTCT	156 bp
Bcl-2	F: TCTTTGAGTTCGGTGGGGTC<br>R: GTTCCACAAAGGCATCCCAG	153 bp
Cytochrome c	F: CCAGGCTGCTGGATTCTCTT<br>R: GGTCTGCCCTTTCTCCCTTC	158 bp
GAPDH	F: AAGTTCAACGGCACAGTCAAGG<br>R: CATACTCAGCACCAGCATCACC	121 bp

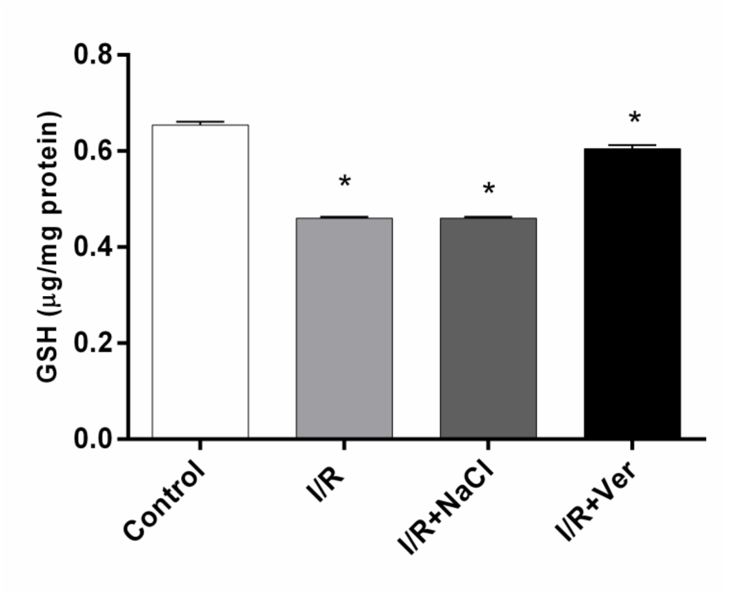

Fig. S1. The GSH content in isolated prefrontal cortex mitochondria.

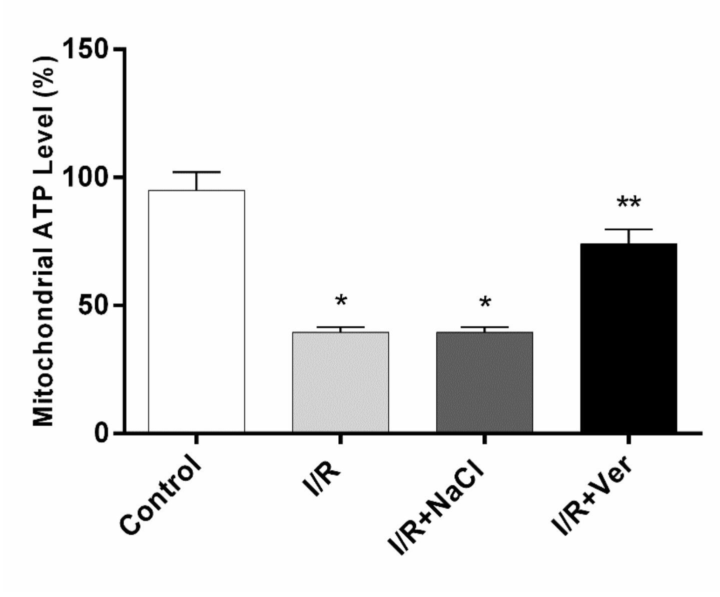

Fig. S2. The ATP levels in the isolated prefrontal cortex mitochondria were determined using Luciferin /Luciferase Enzyme System.

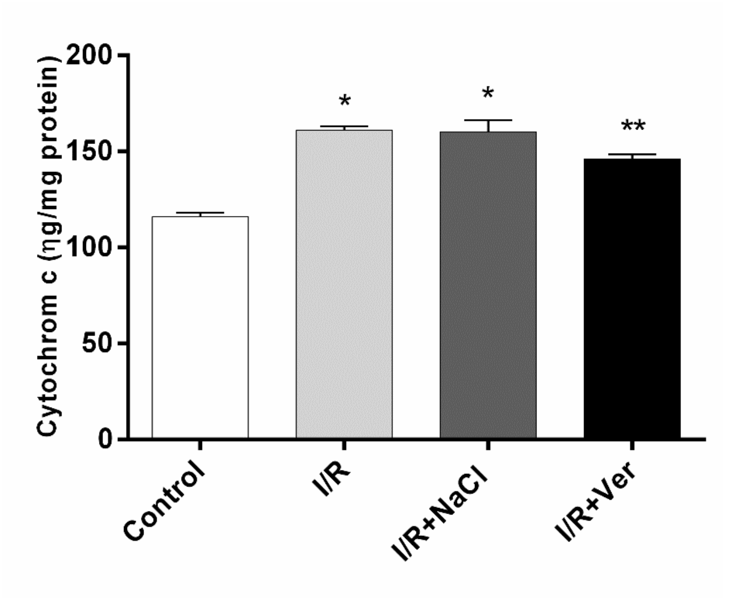

Fig. S3. The Cytochrome c release in the isolated prefrontal cortex mitochondria was measured by ELISA kit.

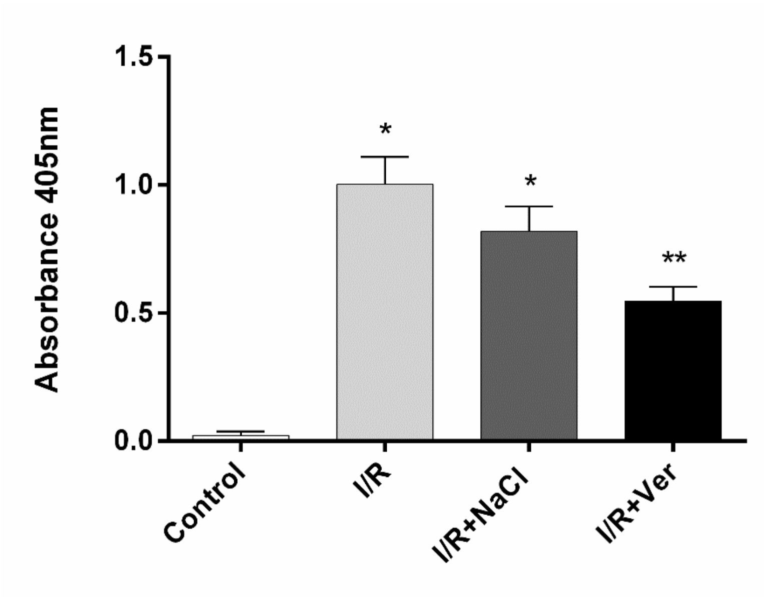

Fig. S4. Effect of the verapamil on the Caspase-3 activity in the prefrontal cortex of rats

Graphical Abstract

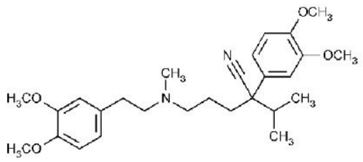

Verapamil (Ver)

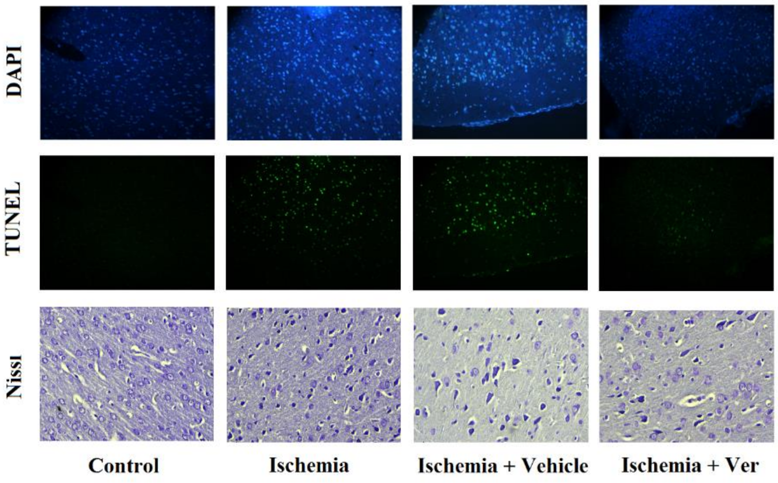

Supplement: Supplementary Materials — Table S1: details of primers (forward and reverse) for target genes used for RT-PCR analysis. Fig. S1: the GSH content in isolated prefrontal cortex mitochondria. Fig. S2: the ATP levels in the isolated prefrontal cortex mitochondria were determined using luciferin/luciferase enzyme system. Fig. S3: the cytochrome c release in the isolated prefrontal cortex mitochondria was measured by ELISA kit. Fig. S4: effect of the verapamil on the caspase-3 activity in the prefrontal cortex of rats. [file 5872645.f1.pdf]
